# Bringing You Negative Selection, Live and in Color

**DOI:** 10.1371/journal.pbio.1001567

**Published:** 2013-05-21

**Authors:** Caitlin Sedwick

**Affiliations:** Freelance Science Writer, San Diego, California, United States of America

T cell activity is driven by a specialized surface receptor called the T cell receptor (TCR), which responds to antigens derived from protein fragments. TCR genes are encoded within a number of gene cassettes that can be rearranged in different combinations to generate TCRs of different antigen specificities. These rearrangements take place as new T cells develop within the thymus, and once a T cell arranges a TCR that successfully recognizes an antigen, TCR gene rearrangement stops and the cell henceforth expresses only that TCR. But not all possible arrangements are useful ones. TCR genes often rearrange in such a way that the resulting TCR recognizes normal body antigens, which could lead to autoimmunity. To prevent autoimmunity, developing T cells undergo a process called negative selection, wherein strongly “self-reactive” T cells are provoked to undergo apoptosis (cellular suicide) before they leave the thymus. While the major steps and molecular mechanisms of negative selection are known, it's proven difficult to study the timing and coordination of these events because they take place within a complex tissue deep inside the body. Ivan Dzhagalov, Ellen Robey, and colleagues set out to address this problem in their paper published in this week's *PLOS Biology*.

**Figure:**
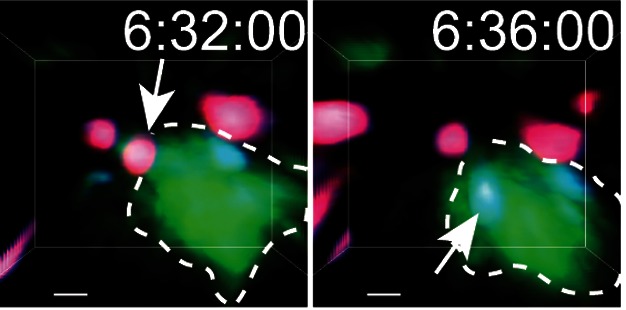
Two snap shots of a thymocyte just before and just after its demise due to negative selection (indicated by red to blue color change). The thymocyte is already enclosed, engulfed by a phagocyte (green) before its death. Time after negative selection stimulus is indicated. Image credit: Ivan L. Dzhagalov and Ellen A. Robey.

The antigens that cause negative selection aren't just floating freely within the thymus; instead, they're presented to T cells by other thymic-resident cells, including dendritic cells, phagocytic cells, and thymic epithelial cells. To test their TCRs for self-reactivity, T cells must migrate about in the thymus seeking out as many contacts as possible with these other cells. A T cell that finds and reacts strongly to a self-antigen experiences robust activation of signaling through its TCR. This strong signaling sends it into apoptosis, a process in which proteases called caspases become activated and destroy a number of essential cellular proteins, DNA fragmentation occurs, and the cell loses its plasma membrane integrity as it dies.

Technical limitations have heretofore forced researchers to study negative selection mainly in single-cell suspensions of dissociated thymic tissue, but Dzhagalov et al. were interested in how T cells behave during negative selection in the intact thymus. They decided to see if the first stage of negative selection (T cell activation) could be detected in slices of intact thymic tissue. For their studies, the authors used transgenic mice whose T cells all express the same TCR, a specially engineered one called F5 TCR that's known to cause negative selection when stimulated with its cognate antigen (which isn't normally present in the thymus). Dzhagalov et al. found that they could synchronously stimulate activation in the F5 T cell population by pipetting the antigen onto slices of thymic tissue.

Having shown that it is possible to activate T cells within thymic slices, the authors then wanted to watch individual T cells undergoing activation. To do this, they used two-photon microscopy to observe F5 T cells expressing green fluorescent protein. As expected, unstimulated F5 T cells were observed to migrate freely within the thymus. But when the authors added in antigen, within a couple of minutes the cells arrested their movement and simultaneously experienced a strong influx of calcium (which is evidence of TCR signaling). Both migration arrest and calcium flux seemed to occur on an all-or-nothing basis in developing F5 T cells—behavior similar to that observed in adult T cells under optimal activating conditions.

As apoptosis is expected to follow activation of F5 T cells, Dzhagalov et al. next looked for signs of apoptosis among F5 T cells in thymic slices. Using antibodies and dyes to monitor the evolution of apoptotic events, the researchers could detect caspase activation and subsequent apoptotic phenomena taking place within the thymus within a few hours after antigen addition. But they were surprised to see that, in contrast with their all-or-nothing behavior with respect to activation and migratory arrest, T cells could delay entry into the apoptotic program. Individual T cells started undergoing apoptosis at erratic intervals after stimulation, and only completed the process after first having made contact with—and sometimes becoming engulfed by—a phagocyte capable of clearing away their corpses. The authors suggest this forbearance may help prevent inflammation caused by release of cellular contents into the tissue.

These data paint some of the finer details into our picture of negative selection, while simultaneously highlighting exciting new areas that need a closer look. For example, since apoptosis is not immediate, can T cells delay the decision to die in hopes of receiving a countermanding signal? This is just one of the questions Dzhagalov and colleagues plan to investigate in the future.

**Dzhagalov IL, Chen KG, Herzmark P, Robey EA (2013) Elimination of Self-Reactive T Cells in the Thymus: A Timeline for Negative Selection. doi:10.1371/journal.pbio.1001566**

